# Supplementary material for: RNA structure prediction using positive and negative evolutionary information
Source: PLoS Comput Biol. 2020 Oct 30;16(10):e1008387. doi: 10.1371/journal.pcbi.1008387 (PMC7657543; doi:10.1371/journal.pcbi.1008387)
Supplement: S1 File — Includes alignments, and source code. (GZ) [file pcbi.1008387.s008.tar.gz › supplemental_material/src/rscape_v1.5.4.tar.gz!/rscape_v1.5.4/lib/infernal/infernal-1.1.2/documentation/userguide/Figures/stl9-63.pdf]

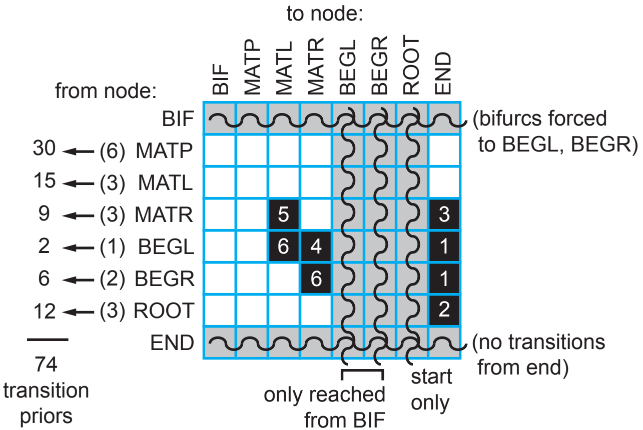

to node:
BIF
MATP
MATL
MATR
BEGL
BEGR
ROOT
END
from node:
BIF
30 ← (6) MATP
15 ← (3) MATL
9 ← (3) MATR
2 ← (1) BEGL
6 ← (2) BEGR
12 ← (3) ROOT
END
74
transition
priors
5
3
6
4
1
6
1
2
(bifurcs forced
to BEGL, BEGR)
(no transitions
from end)
only reached
from BIF
start
only

STL9/63
